# A Genetic and Metabolomic Perspective on the Production of Indole-3-Acetic Acid by *Pantoea agglomerans* and Use of Their Metabolites as Biostimulants in Plant Nurseries

**DOI:** 10.3389/fmicb.2020.01475

**Published:** 2020-07-14

**Authors:** Francesca Luziatelli, Anna Grazia Ficca, Paolo Bonini, Rosario Muleo, Lorenzo Gatti, Massimiliano Meneghini, Michele Tronati, Francesca Melini, Maurizio Ruzzi

**Affiliations:** ^1^Department for Innovation in Biological, Agro-food and Forest systems (DIBAF), University of Tuscia, Viterbo, Italy; ^2^Next-Generation Agronomics (NGA) Laboratory, Tarragona, Spain; ^3^Department of Agricultural and Forestry Sciences (DAFNE), University of Tuscia, Viterbo, Italy; ^4^Vivai Piante Battistini soc. agr. s.s., Cesena, Italy; ^5^Council for Agricultural Research and Economics (CREA), Research Centre for Food and Nutrition, Rome, Italy

**Keywords:** plant growth promotion, auxin, *Pantoea*, indole-3-pyruvate decarboxylase (*ipdC*) gene, culture condition, Q-TOF LC/MS, *Prunus* rootstock, *Corylus avellana* L.

## Abstract

The species *Pantoea agglomerans* includes strains that are agronomically relevant for their growth-promoting or biocontrol traits. Molecular analysis demonstrated that the IPDC pathway involved in the conversion of tryptophan (Trp) to indole-3-acetic acid (IAA) is highly conserved among *P. agglomerans* strains at both gene and protein levels. Results also indicated that the promoter region controlling the inducible expression of *ipdC* gene differs from the model system *Enterobacter cloacae*, which is in accordance with the observation that *P. agglomerans* accumulates higher levels of IAA when cells are collected in the exponential phase of growth. To assess the potential applications of these microorganisms for IAA production, *P. agglomerans* C1, an efficient auxin-producer strain, was cultivated in 5 L fermenter so as to evaluate the effect of the medium formulation, the physiological state of the cells, and the induction timing on the volumetric productivity. Results demonstrated that higher IAA levels were obtained by using a saline medium amended with yeast extract and saccharose and by providing Trp, which acts both as a precursor and an inducer, to a culture in the exponential phase of growth. Untargeted metabolomic analysis revealed a significant effect of the carbon source on the exometabolome profile relative to IAA-related compounds and other plant bioactive signaling molecules. The IAA-enriched metabolites secreted in the culture medium by *P. agglomerans* C1 were used as plant biostimulants to run a series of trials at a large-scale nursery farm. Tests were carried out with *in vitro* and *ex vitro* systems following the regular protocols used for large-scale plant tree agamic propagation. Results obtained with 4,540 microcuttings of *Prunu*s rootstock GF/677 and 1,080 plantlets of *Corylus avellana* L. showed that metabolites from strain C1 improved percentage of rooted-explant, number of adventitious root formation, plant survival, and quality of plant as vigor, with an increase in the leaf area between 17.5 and 42.7% compared to IBA-K (indole-3-butyric acid potassium salt)–treated plants.

## Introduction

Indole-3-acetic acid (IAA) is the most abundant member of the auxin family of phytohormones, and its biosynthesis in plants and bacteria proceeds through distinct biosynthetic routes: both tryptophan (Trp)–dependent and Trp-independent pathways have been described (Eckardt, 2001; Ljung et al., 2002). Based on the distinct intermediates involved in Trp-dependent IAA biosynthesis, five different pathways have been characterized in bacteria, namely, indole-3-acetamide (IAM), indole-3-pyruvic acid (IPyA), indole-3-acetonitrile (IAN), tryptamine (TAM), and Trp side-chain oxidase pathway (Patten et al., 2013; Doktycz, 2019). Although the Trp-independent pathway is thought to occur in bacteria as well (Prinsen et al., 1993), no specific enzymes in this pathway have been characterized.

The IPyA pathway is operational in plant-beneficial bacteria, such as *Azospirillum brasilense* and representative members of *Enterobacter cloacae* complex and is subjected to extremely tight regulation. In this pathway, there is transamination of Trp to IPyA, followed by decarboxylation to indole-3-acetaldehyde (IAAld) by the enzyme indole-3-pyruvate decarboxylase (IPDC; EC 4.1.1.74), and then oxidation of IAAld to IAA. The key enzyme in this pathway, IPDC, is encoded by *ipdC*, and deletion or functional inactivation of *ipdC* gene affects IAA biosynthesis in some strains, such as *Enterobacter ludwigii* (formerly, *E. cloacae*) UW5, *A. brasilense*, *Pantoea agglomerans* 299R, and *Pantoea* species YR343 (Koga et al., 1991; Brandl and Lindow, 1997; Malhotra and Srivastava, 2008; Garcia et al., 2019). The IPDC genes code for polypeptides of approximately 550 amino acids in length, corresponding to a molecular mass of 60 kDa per subunit. The homotetrameric IPDC from *E. ludwigii* UW5, characterized both at biochemical and structural levels, has a molecular mass of 240 kDa and binds four molecules of the cofactors thiamine diphosphate (ThDP) and Mg^2+^ (Schutz et al., 2003). The *ipdC* gene from *E. ludwigii* UW5 is activated by the transcription factor TyrR and increases in response to the aromatic amino acid Trp, tyrosine, and phenylalanine (Ryu and Patten, 2008; Coulson and Patten, 2015). However, regulation varies across bacterial species: constitutive in *Agrobacterium*; regulated by specific transcriptional factors, such as RpoS or RpoN, in some *Pseudomonas* and *Enterobacter* strains; regulated by the global signal transduction system GacS/GacA that controls secondary metabolism in several plant-associated gram-negative bacteria (Spaepen et al., 2007).

Indole-3-acetic acid plays an important role in the regulation of growth and development of vascular plants, including cell division, cell extension, and cell differentiation (Kasahara, 2016). It specifically plays a crucial role in root initiation, apical dominance, tropisms, and senescence (Zhao, 2010). In addition, IAA is produced by plants, as well as by some beneficial bacteria in the rhizosphere, where it acts as a signaling molecule with significant effects on the communication between plants and microorganisms, and on plant growth (Spaepen and Vanderleyden, 2011; Duca et al., 2014).

In recent years, several studies have reported alternative approaches to the application of phytohormones and plant growth regulators, such as the use of symbiotic organisms and/or natural biostimulants from microbial and non-microbial organisms in agriculture systems and in tissue cultures, which are also environment friendly, as for microbial biostimulants (Ruzzi and Aroca, 2015; Orlikowska et al., 2017; Rouphael and Colla, 2018). Within this framework, the species *P. agglomerans* has drawn attention for its plant growth–promoting activity.

Classification of *P. agglomerans* into the biosecurity group 2 and the fact that sometimes this species causes human infections particularly in immunocompromised people prevent its utilization as bioinoculant in Europe (Dutkiewicz et al., 2016a; Büyükcam et al., 2018). However, increasing evidence has shown that selected members of the *P. agglomerans* species can have a great potential as plant growth–promoting bacteria (Paredes-Páliz et al., 2016a, b, 2017) and comprise strains that are agronomically relevant for their growth-promoting or biocontrol traits and have been increasingly regarded as ideal candidates among plant growth–promoting rhizobacteria to be used as a biocontrol agent (Dutkiewicz et al., 2016b).

In details, *P. agglomerans* strain C1, isolated from the phyllosphere of lettuce plants (*Lactuca sativa* L.) treated with plant-derived protein hydrolysates (Luziatelli et al., 2016), has been studied for its potential as novel biostimulant in sustainable agriculture. It has been specifically characterized for its heavy metal resistance and metabolic capacities (Luziatelli et al., 2019b, 2020), as well as for its ability to solubilize phosphate, to inhibit plant pathogens, to produce IAA and siderophores, and to improve the use of rock phosphates and the growth of corn (*Zea mays* L.) and tomato (*Solanum lycopersicum* L.) in pot experiments (Luziatelli et al., 2019a, 2020; Saia et al., 2020). In particular, when *P. agglomerans* strain C1 is grown onto medium rich in Trp, large amounts of IAA are produced, which makes it a natural biostimulant suitable to be used in the rooting phase of micropropagation instead of the synthetic auxins (Luziatelli et al., 2020).

Micropropagation allows a rapid multiplication of several plant species in large-scale clonal plants and is becoming a widespread technique for rootstocks and crops species propagation (Loberant et al., 2010). On the nursery farming scale, micropropagation has a strong economic impact, because in a relatively short time period, reduced space and growing controlled conditions are required independently of environmental conditions and season period (Akin-Idowu et al., 2009). Micropropagation starts, in fact, from a small part of selected elite plant and allows the production thousands of plants that can be produced in a continuous process (Pierik and Scholten, 1994). However, rhizogenesis and root growth are critical morphophysiological events for plant survival in *ex vitro* conditions and are achieved by auxin treatment of unrooted microshoots as the last phase of *in vitro* culture and/or the first phase of *in vivo* acclimatization phase.

In micropropagation of woody crops, it has been observed that, besides IAA, synthetic molecules with strong auxin activities can be used. Among them, indole-3-butyric acid (IBA) is the compound of choice, thanks to its higher stability in the culture medium (Nissen and Sutter, 1990; Bhatti and Jha, 2010). Moreover, it has been assumed that, among the metabolites making up the pool of excreted molecules (exometabolome), there are molecules that could have a synergistic activity with auxin and/or elicitor roles, acting on the regulation pathways of adventitious rooting events and quality of roots.

The study was designed to test the biostimulant properties of metabolites excreted by *P. agglomerans* strain C1 on root induction and plant development of some woody fruit crop species in large-scale nurseries, at the phase of *in vitro* and *ex vitro* rooting and acclimation.

## Materials and Methods

### Strain and Media

*Pantoea agglomerans* C1 is an environmental strain isolated from the phyllosphere of lettuce (Luziatelli et al., 2016, 2019a). Strain C1 was maintained as LB Lennox (Lennox, 1955)–glycerol 20% (vol/vol) stocks at −80°C and revived on LB broth at 30°C under agitation [180 revolutions/min (rpm)]. For production of IAA, the strain can be cultivated on both saline and rich media supplemented with Trp (4 mM). A total of six media were used: LB broth Lennox (per liter, tryptone 10 g, yeast extract 5 g, NaCl 5 g); saline M9-glucose medium (Maniatis et al., 1982); yeast extract sucrose (YES) and yeast extract glucose (YEG) medium [1 × M9 saline solution, yeast extract 5 g L^–1^, carbon source (sucrose or glucose), 5 g L^–1^]; and reinforced YES/YEG medium (rYES and rYEG medium containing double the amount of yeast extract, 10 g L^–1^).

### Pathway and Gene Identification

As a preliminary step, a database of bacterial proteins that are associated to the five major IAA biosynthetic pathways was constructed following a comprehensive search of the literature. Then, the complete set of 4497 protein sequences encoded by *P. agglomerans* C1 (Luziatelli et al., 2020) was compared against this manually curated set of IAA biosynthetic genes using the Basic Local Alignment Search Tool (BLAST; Altschul et al., 1990). Multiple protein sequence alignments were generated using ClustalW algorithm (Thompson et al., 1994). Bacterial promoter prediction program, BPROM^1^, was used to identify the position of the promoter, that is, -10 box and -35 box in the input sequence. The PATRIC BLASTN and BLASTP services were used to perform homology searches against *P. agglomerans* genomes available at PATRIC Website^2^ using, as queries, nucleotide/protein sequences of strain C1 corresponding to (1) the 4 kb *ipdC*-containing region; (2) the 5′ upstream region of *ipdC* gene; (3) the genes encoding aminotransferase (peg.1678), IPDC (*ipdC*; peg.1955), and IAAld dehydrogenase (peg.576 and peg.879); (4) the deduced protein sequences of peg.1678, peg.1955, peg.576, and peg.879. The resulting hits were filtered at 100% query coverage and ≥ 80% of sequence identity.

### Growth Conditions

Seed cultures, from a freeze glycerol stock of *P. agglomerans* strain C1, were inoculated into 500 Erlenmeyer flasks containing 50 mL of LB medium and incubated at 30°C with agitation (180 rpm).

Seed cultures in the late exponential phase of growth [optical density at 600 nm (OD_600_) of 4.5–4.8] were used to inoculate, with an initial OD_600_ of 0.1, 25 mL of production medium amended with Trp (4 mM), and growth was monitored by measuring the wet weight of cells. In addition, LB + Trp (4 mM) was used as reference media for IAA biosynthesis.

After 24 h of growth at 30°C and 180 rpm, cells were removed by centrifugation (10 min at 8,000 g), and the supernatant was filter-sterilized through a 0.22 μm filter and stored at -20°C until use.

### Fermentation and Optimization of the Induction Conditions

To evaluate the relationship between the physiological state of the cells and the IAA production level, *P. agglomerans* strain C1 was grown in a BioFlo 120 bench-top stirred tank reactor (Eppendorf S.r.l., Milan, Italy) equipped with a 7.5-L vessel, two Rushton impellers, digital ISM probes for dissolved oxygen (DO), and pH (Mettler-Toledo S.p.A., Milan, Italy) and a platinum RTD (Pt100) sensor for the temperature. The growth was carried out in YES medium (5 L) at 30°C under aerobic conditions. The oxygen concentration was maintained greater than 20% of saturation (oscillation between 25 and 40%), blowing sterile air at an aeration rate of 0.2–1.5 (vol/vol/m), and by regulating the impellers speed from 150 rpm (initial condition) to 525 rpm (end of the exponential phase of growth). The initial pH of the medium was adjusted to 6.6 by addition of 1 M HCl or 2 M of NaOH, and growth was carried out without pH control.

The reactor was inoculated at 5% (vol/vol) with an initial optical density (OD_600_) of 0.4, using an LB culture grown at 180 rpm and 30°C up to the late exponential phase. After 3, 3.5, 4, 4.5, 5, and 6 h of growth, 100 mL aliquots were collected from the fermenter, and the cells recovered by centrifugation (8,000 rpm for 10 min) were resuspended in fresh YES medium to obtain a final OD_600_ of 10 and used to inoculate shaken flasks containing YES medium (25 mL) amended with Trp (4 mM). All cultures were inoculated at the same initial OD_600_ of 0.5 and incubated at 180 rpm and 30°C for 18 h. For each time point, experiments were carried out in triplicate. To measure the accumulation of IAA in the culture medium, 1 mL samples were taken at intervals of 60 min for the first 2 h and at the end of the growth (18 h) and treated as reported before.

### Spectrophotometric Determination of Indole Auxins

Auxin production was measured using Salkowski reagent as described previously (Luziatelli et al., 2020). In brief, 1 mL of filter-sterilized (0.22 μm) supernatant was added to 2 mL of Salkowski reagent (0.5 M FeCl_3_, 35% vol/vol HClO_4_), and the mixture was incubated at room temperature (in the dark) for 20 min. The presence of IAA and other indole auxins were detected measuring pink color development at 535 nm using a Cary 50 UV-Vis spectrophotometer (Agilent, Santa Clara, CA, United States). A series of IAA standard solutions of known concentrations were prepared to set up the calibration curve.

### Metabolome Analysis by Quadrupole Time-of-Flight Liquid Chromatography–Mass Spectroscopy

For untargeted metabolomics, the sample (1 mL) was extracted in 5 mL of cold (−20°C) acidified (0.1% HCOOH) 80/20 methanol/water using an Ultra-Turrax Homogenizer (Ika T-25, Staufen, Germany), centrifuged at 1200 rpm and filtered through a 0.2 μm cellulose membrane. The analysis was carried out on an Agilent 6550 Q-TOF with ESI source, coupled with an Agilent 1290 UHPLC. A BEH C18 column from Waters (100 × 2.1 mm internal diameter, 1.7 μm) was used according to the procedure and gradient described in Tsugawa et al. (2019). Injection volume was 2 μL for all samples. A pooled quality control was obtained by mixing 10 μL of each sample and acquired in tandem mass spectroscopy mode using iterative function five consecutive times to increase the number of compounds with associate MS2 spectra. Blank filtering, alignment, and identification were accomplished using MS-DIAL (Tsugawa et al., 2015) and MS-FINDER (Tsugawa et al., 2016), with the procedure described by Blaženović et al. (2019).

The table with all compound peaks height was exported from MS-DIAL into MS-FLO (DeFelice et al., 2017) to reduce false positives and duplicates. Then, an internal developed workflow in R was employed for fold change and Benjamini-Hochberg corrected *p*-value, PLSDA analysis (Thévenot et al., 2015), and chemical enrichment analysis (Barupal et al., 2012).

### Plant Inoculation

*In vitro* shoot tips of peach clonal rootstock “GF677” (*Prunus persica* × *Prunus amygdalus*) of 10 mm in length and *in vitro* shoot tips of hazelnut cv. “Fertile de Coutard” (*Corylus avellana* L.) of 15 mm in length were cultured on proliferation medium, containing a modified Quirin and Lepoivre (QL; Quoirin and Lepoivre, 1977) and Driver and Kuniyaki Walnut (DKW; Driver and Kuniyuki, 1984) basal salt solution, respectively, and enriched by 30 g L^–1^ sucrose and solidified by 6.8 g L^–1^ agar. The growth regulators added to the medium were as follows: 2.22 μM of 6-benzyladenine (BA), 0.05 μM of α-naphthalene acetic acid for the rootstock GF677; 10 μM of BA, 0.05 μM of IBA for the cv. Fertile de Coutard. The pH of the medium was adjusted to 6.3 ± 0.1 for the rootstock GF677, and 6.0 ± 0.1 for the cv. Fertile de Coutard, before addition of agar and sterilization at 120°C for 20 min. Shoots of rootstock GF677 were subcultured at a 4-week interval, while shoots of cv. Fertile de Coutard were subcultured at a 6-week interval, under 16 h light photoperiod, using white fluorescent lamps Philips TL-D 58/865-MASTER (Philips, Italia), at 40 μM m^–2^ s^–1^ photon flux at constant temperature of 23°C ± 1°C.

After the proliferation step, microcuttings were transferred for 15 days in Murashige and Skoog (MS) elongation medium (Murashige and Skoog, 1962), supplemented with 14 μM gibberellic acid (GA3) and 20 g L^–1^ sucrose. The medium was sterilized at 120°C (2 bars), for 20 min after addition of 6.8 g L^–1^ agar and pH titration to 6.5. Glass jars of 500 mL in volume, each containing 100 mL of culture medium, were used as culture vessels.

At the beginning of the acclimatization, before the transfer into the cell plug tray, plantlets were immersed for 10 s in an auxin solution containing either an appropriate dilution of IAA-enriched excretome from *P. agglomerans* strain C1 (indole auxins final concentration of 1 μM) or 10 μM IBA potassium salt (IBA-K). *Ex vitro* acclimatization started on April 2018 by transferring the treated plantlets into 360 cells plug tray (Jiffy, Netherlands), with a volume of each cell cavity of 7 cm^3^.

Trays were placed under controlled misting of the greenhouse (temperature 28°C ± 4°C, RH 90%), at natural photoperiod and photosynthetic active radiation varying daily between 300 and 500 μM m^–2^ s^–1^.

All on-farm trials, two with *Prunus* rootstock GF677 and one with hazelnut, were carried out in agreement with the company’s production cycle. In the first trial with rootstock GF677, 720 plants (two trays of 360 cells) were treated by dipping with secreted metabolites from strain C1, and an equal number of plants were treated with IBA-K solution. In the second trial, 3,780 plants (10.5 trays of 360 cells) were treated with C1 metabolites, and 1,800 plants (five trays of 360 cells) with IBA-K. In both experiments, after 10 days from the dip treatment, plants were treated again by spraying fine drops of the same solution until the complete wetting of the leaves was achieved.

For hazelnut adventitious rooting induction experiments, 1,440 plantlets were treated by dipping with secreted metabolites from strain C1, and 1,080 plantlets were treated with IBA-K.

On the 20th day of the *ex vitro* acclimatization, the percentage of rooted plantlets, the number of roots per plantlet, the root length, and the elongation of plantlet stem were recorded.

After 1 month, all plantlets were transferred into 60 preloaded cells plug tray (Jiffy), each cell plug with a volume of 117 cm^3^. Two weeks later, the survival ratio of plant was determined, and the total leaf area per plant was detected by Android “CANOPEO” application^3^ on a total of 600 randomly chosen plants (300 for control and 300 *Pantoea*-treated).

Since the beginning of the transfer *in vivo*, every 20 days, plants were fertilized with NUTRIGREEN AD (GREEN HAS ITALIA S.p.A.), through the fertigation system at the amount of 2 mL L^–1^.

### Statistical Analysis

Statistically significant differences between the means were determined by the one-way analysis of variance using the SigmaStat 3.1 package (Systat Software Inc., San Jose, CA, United States).

## Results

### Identification of the IAA-Biosynthetic Genes

**FIGURE 1:**
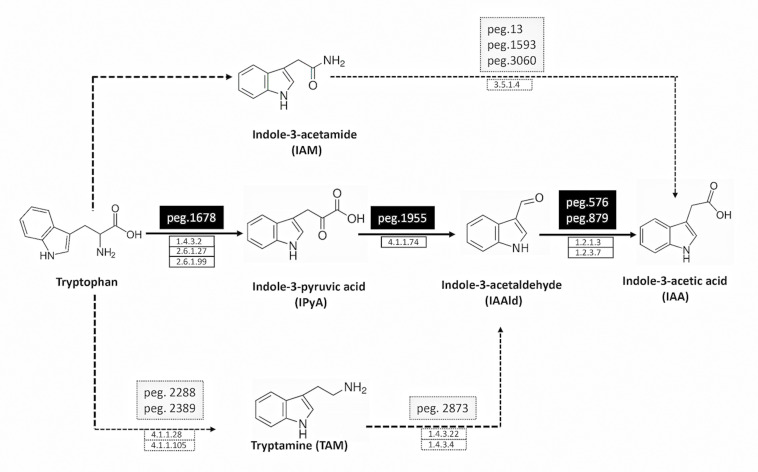
Deduced pathways for IAA biosynthesis in *P. agglomerans* strain C1. The solid line indicates the principal pathway for IAA biosynthesis, and the dotted ones the pathways that are not fully supported by molecular evidences (low identity with known proteins; see Table 1 for details). The names of the pathways indicate the name of their first products. The names of the protein encoding genes (peg) and the definition of the protein coding enzymes (EC number) are reported on the top and the bottom of the arrows, respectively.

Genes encoding enzymes involved in IAA synthesis were identified in *P. agglomerans* C1 genome by BLASTx analysis using the sequences of 11 different enzymes associated with alternative IAA pathways, as queries (Table 1). This analysis revealed the presence, in strain C1, of the whole set of genes of only one of the five IAA pathways occurring in bacteria and plants: the indole-3-pyruvic acid (IPyA) pathway (Table 1 and Figure 1). Noteworthy, for each of the three enzymes involved in this pathway, the identity, at amino acid level, with sequences of other *Pantoea* strains was between 81 and 92% along the entire protein length (Table 1). For the same proteins, the identity between sequences from strain C1 and strains belonging to other genera, including *Azospirillum*, *Pseudomonas*, *Enterobacter*, *Azospirillum*, and *Arthrobacter*, varied between 25 and 56% (Table 1).

**TABLE 1:** Identification of protein encoding genes (peg) and enzymes involved in IAA biosynthesis in *P. agglomerans* strain C1 based on a systematic computational approach.

**Pathway**	**Enzymes**	**Query source**	**C1 representatives**	**Template sequence identity/coverage (%/%)**
IPyA	Aminotransferase (EC 1.4.3.2) (EC 2.6.1.27) (EC 2.6.1.99)	> AEB97257.1 HisC1 [*Azospirillum brasilense* Sp7]^§^	peg.1678	28/91
		>PMI_01811_Am_Trf1[*Pantoea* speciesYR343]^§^		88/100
	Indole-3-pyruvate decarboxylase (EC 4.1.1.74)	> AAB06571 [*Pantoea agglomerans*_299R]^§^	peg.1955	92/100
		>PMI_00059_IPDC1 [*Pantoea* speciesYR343]^§^		73/100
		>AKM88529[*Enterobacter ludwigii* UW5, formely *E. cloacae* UW5]^§^		56/99
		>AAG00523 [*Pseudomonas putida* GR12-2]^§^		56/99
		>BAA14242 [*Enterobacter cloacae* FERM BP-1529]^§^		56/94
		>AAC36886 [*A. brasilense* Sp245]^§^		27/92
		>ABV24338[*Paenibacillus polymyxa* E681]^§^		25/80
	Indole-3-acetaldehyde dehydrogenase (EC 1.2.1.3) (EC 1.2.3.7)	> WP_040136836.1 [*A. brasilense*]^§^	peg.1406	42/96
		> AAC49575 [*Ustilago maydis*]^§^	peg.766	42/94
		> NP_789951[*Pseudomonas syringae* DC3000]^§^	peg.1613	41/95
		> NP_792480 [*P. syringae* DC3000]^§^	peg.3848	40/96
		> WP_042703524.1[*Azospirillum* species B506]	peg.1614	39/94
		> ELT45240.1 [*Arthrobacter nitrophenolicus* SJCon]	peg.1183	36/96
		> PMI39_00794 IALDH7[*Pantoea* speciesYR343]^§^	peg.879	81/100
		> PMI39_04236 IALDH16[*Pantoea* speciesYR343]^§^	peg.576	87/100
IPyA-YUCCA	Indole-3-pyruvate monooxygenase	> Q9SZY8.1 YUCCA1 [*Arabidopsis thaliana*]	–	–
		>BAS07733.1 YUCCA3 [*Arthrobacter* species Hiyo4]		
IAM	Trp-monoxygenase	> AGL87350.1 IaaM [*Pseudomonas protegens* CHA0]^§^	–	–
		>AAD30489.1 [*Agrobacterium fabrum* str. C58]^§^		
	Indole acetamide hydrolase (EC 3.5.1.4)	> WP_087872787.1 [*Arthrobacter globiformis*]	peg.1593	34/97
		> P0A2 × 0 [*Agrobacterium tumefaciens* pTiA6 plasmid]^§^	peg.13	27/97
		> AAA17679.1 [*P. syringae* Y30]^§^	peg.3060	40/61
IAN	Oxidoreductase	> O81346.2 [*A. thaliana*]	–	–
		>Q501D8.1[*A. thaliana*]		
	Acetaldoxime dehydratase	> BAA90461.1 [*Bacillus* species OxB-1]	–	–
		>BAD17969.1 [*Rhodococcus erythropolis* N-771]		
	Nitrile hydratase	> AFY20546.1 [*Pseudomonas* species UW4]	–	–
		>AFY20547.1 [*Pseudomonas* species UW4]		
TAM	Trp decarboxylase (EC 4.1.1.28) (EC 4.1.1.105)	> 4OBV_A Chain A [*Ruminococcus gnavus* ATCC29149]^§^	peg.2389	31/80
			peg.2288	30/80
	Amine oxidase (EC 1.4.3.22) (EC 1.4.3.4)	> EZQ09285 [*A. brasilense* Az39]	peg.2873	24/93

**^§^Proteins whose function is supported by biochemical evidences.**

Proteins with weak similarity to (i) indole acetamide hydrolase (pathway IAM) and (ii) Trp decarboxylase and amine oxidase (pathway TAM) were also identified (Table 1 and Figure 1). In contrast, no sequence related to Trp aminotransferase and YUCCA enzymes (pathway IPyA-YUCCA) or nitrilases (pathway IAN) was detected (Table 1).

### Sequence Comparison of IPDC Proteins

**FIGURE 2:**
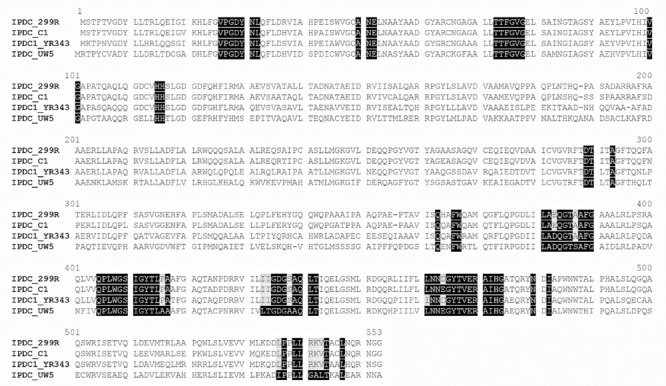
Sequence alignment of peg.1955 encoded protein from *P. agglomerans* strain C1 and functionally characterized IPDC from *E. ludwigii* and other *Pantoea* strains. Residues in IPDC sequence from *E. ludwigii* UW5 that have been identified by crystal structure analysis as being at the active site or involved in either ThDP or substrate binding are highlighted in black. In the alignment, the amino acid residues of these regions that are conserved among all or almost all different sequences are highlighted in black or gray.

BLAST analysis of the ThDP-binding indolepyruvate decarboxylase (IPDC EC 4.1.1.74) revealed (Table 1) that the deduced amino acid sequence encoded by C1_peg1955 from *P. agglomerans* C1 (IPDC*_Pa__*_C__1_) shares a 92% of identity with IPDC from *P. agglomerans* (formerly *Erwinia herbicola*) strain 299R (IPDC*_Pa__*_299__R_; Brandl and Lindow, 1996) and 73% of identity with IPDC1 from *Pantoea* species strain YR343 (IPDC1*_P_*_sp_YR__343_; Garcia et al., 2019). To gain more information about IPDC*_Pa__*_C__1_, a comparative analysis was carried out between this protein and IPCD from *E. ludwigii* (previously misidentified as *Pseudomonas putida* and subsequently as *E. cloacae*) strain UW5 (IPDC*_Ec_*__UW__5_). The structural model of IPDC*_Ec_*__UW__5_ has been published almost 10 years ago by Schutz et al. (2003), and the authors demonstrated that IPDC*_Ec_*__UW__5_ is a homotetrameric enzyme in which each monomer has defined domains involved in the binding of both the substrate and the cofactors (Mg^2+^ and ThDP). Using the MULTALIN tool (Corpet, 1988), IPDC sequences from strain C1, *E. ludwigii* UW5 and other *Pantoea* strains were aligned as reported in Table 1. This analysis allowed demonstrating that several amino acids, which were found to be essential for the activity of IPDC*_Ec_*__UW__5_, were conserved or conservatively replaced in all *Pantoea* strains (Figure 2). In detail, 90% of the active site residues of IPDC*_Ec_*__UW__5_ are conserved in IPDC*_Pa__*_C__1_, suggesting that the two enzymes might have a similar catalytic mechanism.

### Organization of the IPDC Coding Region

**FIGURE 3:**
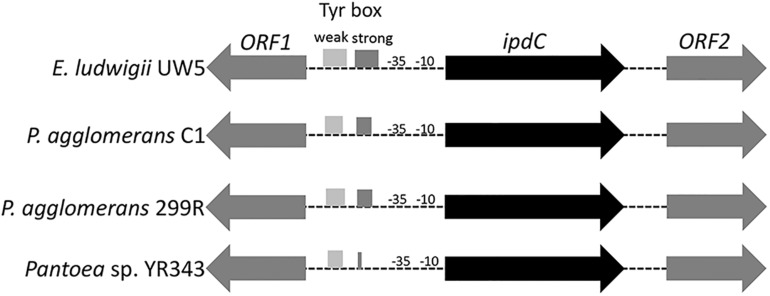
Organization of the chromosome region surrounding *ipdC* across different species. The boxes in the *ipdC* promoter region indicate the location of the sequences with partial similarity to the two TyrR binding boxes occurring in *E. ludwigii* EW5. The predicted -10 and -35 elements for *ipdC* gene are also shown.

**FIGURE 4:**
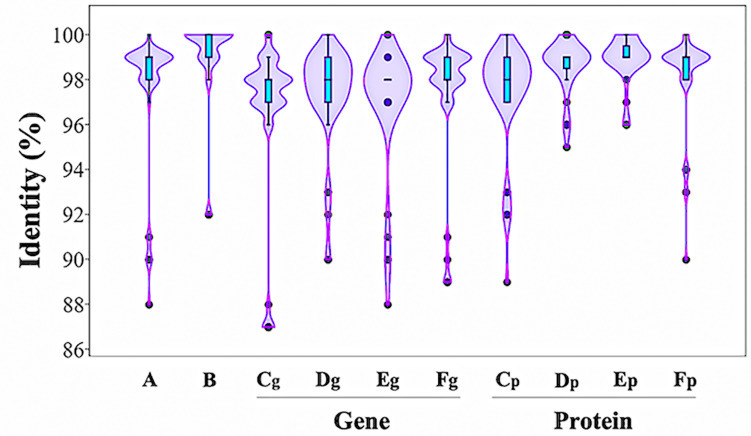
Distribution of sequence identity matches of *P. agglomerans* strain C1 to other *P. agglomerans* DNA and protein sequences related to IPyA pathway. The violin plots show the distribution of sequence identities of the *ipdC* gene cluster (A), the *ipdC* promoter region (B), the IPDC gene (C_g_) and protein (C_p_), the amino transferase gene (D_g_) and protein (D_p_), and the two IAAld dehydrogenase genes (E_g_ and F_g_) and proteins (E_p_ and E_p_).

DNA sequence analysis of the genomic region surrounding the *ipd*C gene from *P. agglomerans* C1 revealed the presence of two ORFs encoding a 330-amino acid protein, annotated as L-glyceraldehyde 3-phosphate reductase (ORF1), and a 322-amino acid protein, with high homology to glucokinase, respectively (Figure 3). Interestingly, a similar genetic structure occurs in *E. ludwigii* UW5 (Coulson and Patten, 2015) and *Pantoea* species YR343 (Garcia et al., 2019; Figure 3), as well as in 45 of 50 *P. agglomerans* genomes sequenced so far. In the latter case, the identity over the entire 4 kb sequence of the ORF1-*ipdC*-ORF2 gene cluster was between 88 and 100%, with a mean value of 99% (Figure 4, lane A). Surprisingly, a high sequence identity (mean value = 98%) was also observed (Figure 4, lane C*g*-F*g*) comparing the DNA sequence of all *P. agglomerans* genes of the IPyA pathway, the genes encoding amino transferase, IPDC and IAAld decarboxylase (peg.1678, peg.1955, peg.576, and peg.879 in strain C1; Figure 1). The remarkable evolutionary conservation of the IPyA pathway among the members of the *P. agglomerans* species was also confirmed analyzing the variability at the level of protein sequence that varied between 1 and 2% and the kernel density plot (Figure 4, lane C*p*-F*p*). A similar observation was done analyzing the *ipdC* promoter region, which, as shown by the density of the data in the violin plot reported in Figure 4 (lane B), is more conserved than the IPCD coding sequence.

### Analysis of the *ipdC* Promoter

**FIGURE 5:**
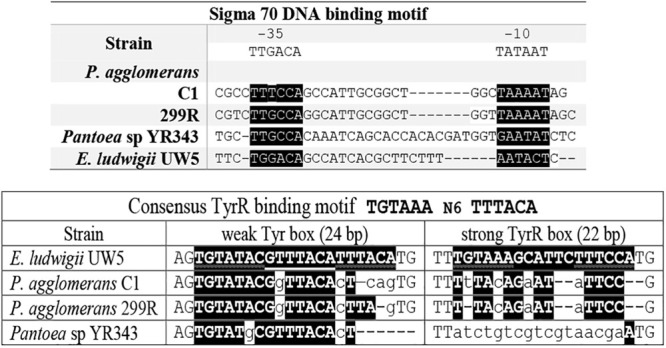
Alignment of relevant elements of the *ipdC* promoter sequence of *E. ludwigii* UW5, *P. agglomerans* C1, *P. agglomerans* 299R, and *Pantoea* species YR343. The conserved nucleotides in the -10 and -35 region (upper panel) and putative TyrR binding sites (lower panel) are highlighted in black.

A search of functional motifs carried out with BPROM annotation package (Softberry Inc., Mount Kisco, NY, United States) revealed the presence, in the promoter region upstream of *P. agglomerans* C1 *ipd*C gene, of sequences that resemble to the *Escherichia coli* RpoD (s^70^) -10 and -35 elements, matching the *E. coli* consensus sequences at four of six nucleotides (-10), and five of six nucleotides (-35), respectively. Interestingly, the *ipd*C putative regulatory sites of C1 showed significant similarity with those predicted in the *ipd*C promoter of other *Pantoea* strains (YR343 and 299R) and *E. ludwigii* UW5 (Figure 5).

Inspection of the 5′ untranslated region upstream from the C1_*ipd*C start codon showed no detectable *cis*-regulatory element such as the inverted repeats identified in the *ipd*C promoter of *A. brasilense* (Vande Broek et al., 2005) or the two 18-bp consensus sequences (weak and strong TyrR boxes) recognized by TyrR, which regulates the expression of the *ipd*C gene in *E. ludwigii* UW5 (Coulson and Patten, 2015). Sequences with weak similarity to the TyrR consensus motif (TGTAAA-N_6_-TTTACA) were found in the *ipdC* promoter region from *Pantoea* strains, but they do not meet the minimum molecular requirements for TyrR-mediated regulation: the presence of a strong TyrR box or a weak box with an adjacent strong box; the presence in the TyrR box of the G-C residues, essential for TyrR binding, spaced 14 bp apart (Figure 5). These evidences strongly support the hypothesis that the transcription of the *ipdC* gene in *P. agglomerans* is not controlled by the TyrR regulatory system.

### Selection of the Growth Medium for IAA Production

In preliminary experiments, it was observed that, in contrast to LB, when *P. agglomerans* C1 cells were grown on saline M9-glucose medium without Trp, no basal level of IAA was produced. Providing Trp (as inducer and precursor) at 4 mM concentration, no significant difference was observed in the IAA final titer (54 ± 0.9 mg L^–1^) shifting from LB to M9-glucose medium. To further investigate the possibility to use a medium that has equivalent performances of LB but is free of animal-derived ingredients, the ability of C1 cells to grow and produce IAA on a simplified culture medium containing a saline phosphate buffer (M9 saline solution), a sugar, as a carbon source, and yeast extract, as a source of organic nitrogen, vitamins, and other growth factors, was investigated. For this purpose, glucose or sucrose, as a carbon source, was used alternatively, in combination with two concentrations of yeast extract (5 or 10 g L^–1^). All four media were amended with Trp (4 mM), and LB-Trp was used as a control.

**FIGURE 6:**
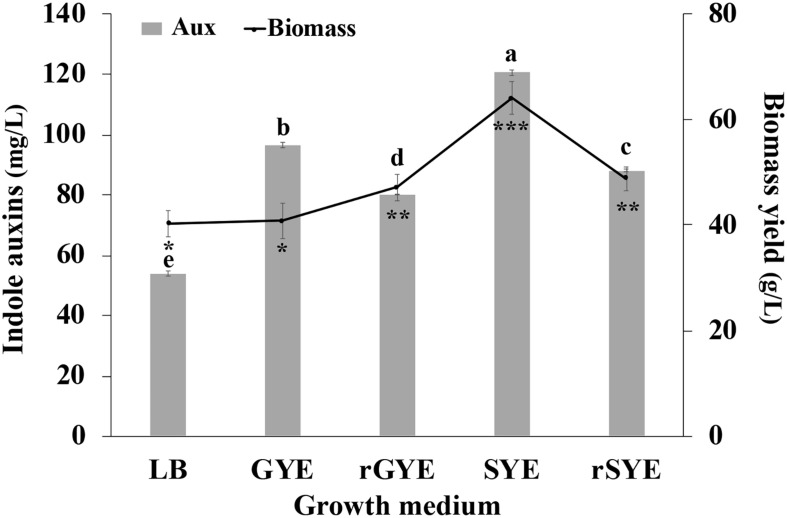
Effect of culture medium on IAA production (mg L^–1^) and biomass concentration (g [wet weight] L^–1^). LB, YEG, and YES contained 5 g L^–1^ of yeast extract; in reinforced media (rYEG and rYES) the concentration of this ingredient is doubled (10 g L^–1^). Differences in letters and symbols indicate that the values are significantly different (*P* > 0.05).

Results reported in Figure 6 indicate that IAA production occurred in all tested conditions and, independently from the carbon source and the amount of yeast extract that was used, was higher in saline medium compared to the control medium (LB-Trp). In particular, the higher level of biomass (64.1 ± 3.1 g [wet weight] L^–1^) and IAA (120.5 ± 0.9 μg mL^–1^) were obtained cultivating the microorganism in the presence of both sucrose and yeast extract at a final concentration of 0.5% (wt/vol) (SYE medium; Figure 5). Surprisingly, an increase in the organic nitrogen content, with double the amount of yeast extract [from 0.5 to 1% (wt/vol)], did not affect IAA production (80.13 ± 0.11 μg mL^–1^ in rGYE and 88.1 ± 0.44 μg mL^–1^ in rSYE) and biomass yield (47.1 ± 2.5 or 48.7 ± 2.2 g [wet weight] L^–1^) (Figure 6).

Interestingly, there was no significant difference in the biomass yield when cells were grown on GYE or LB medium (40.8 ± 3.3 g [wet weight] L^–1^ and 40.3 ± 2.5 g [wet weight] L^–1^), but the IAA-specific productivity shifting from LB to GYE had approximately 1.8-fold increase (Figure 6). These results suggested that LB was not an optimal medium for IAA production by *P. agglomerans* strain C1.

### Overall Impact of the Carbon Source on the Metabolites Secreted by *P. agglomerans* C1

**FIGURE 7:**
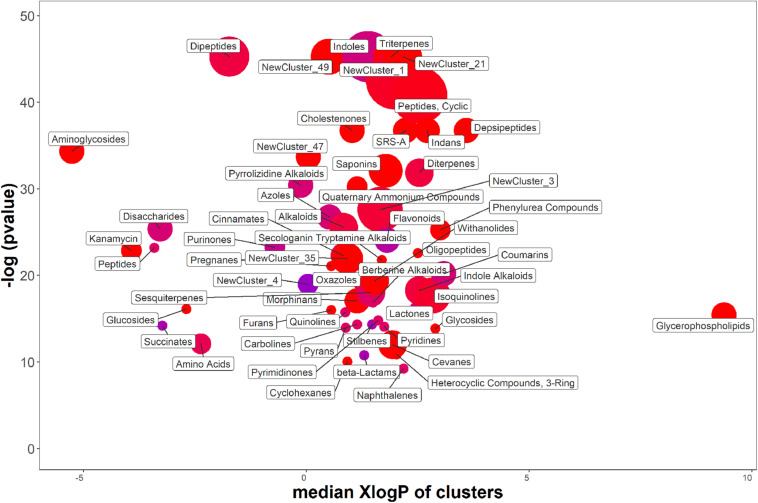
ChemRich analysis of exometabolome of *P. agglomerans* C1 grown on YES vs. YEG medium. Red clusters associated with higher outcomes, and the blue ones associated with lower outcomes.

Results obtained in shake-flask experiments indicated that, in contrast to previous finding obtained with *P. agglomerans* 299R (Brandl and Lindow, 1997), the production of IAA from strain C1 was affected by the carbon source. For this reason, the effect of the carbon source on the production of IAA and IAA-related compounds was studied in more detail by analyzing the exometabolome of *P. agglomerans* C1 using an untargeted metabolomics approach. Of the 528 features detected in the exhausted growth medium, when cells were cultured on YES or YEG amended with Trp, a total of 381 were more than twofold higher, and 94 were more than twofold lower when sucrose was used as a carbon source. ChemRICH analysis showed that a total of 58 metabolite clusters were significantly different (*P* > 0.05) between YES and YEG (Figure 7 and Supplementary Table S1). For 27 of these clusters, the differences arise from an increased level in all the compounds of the cluster, and for other 10 clusters, the enriched molecules represented 75–95% of the total compounds of the cluster. As shown in Figure 7, in sucrose-grown cultures, the classes of metabolites with the highest elevated level were as follows: dipeptides and cyclic peptides; triterpenes; compounds belonging to new clusters 1, 21, and 49. Clustering analysis also revealed significant differences in the concentration level of single metabolites belonging to the indole-oligopeptides clusters and to the flavonoids cluster. In the first two clusters, we observed an increase of some bisindole alkaloids, such as the guaiaflavine (140-fold); an increase of some monoindole alkaloids, such as the indole-3-carbinol (I3C; 4.3-fold) and the IAA (2.2-fold); an increase of some amide-linked-IAA-L-amino acid conjugates (IAA-aa), such as indole-3-acetyl-L-valine (39-fold) and indole-3-acetyl-L-leucine (2.4-fold); a threefold decrease of aldehyde derivatives, such as IAAld and ^1^H-indole-3-carboxaldehyde (I3A) (Supplementary Table S2). In the flavonoids cluster, a significant increase in N-containing flavonoids, such as phyllospadine (54-fold increase), was observed. These results clearly indicate that in *P. agglomerans* C1 the levels of secreted metabolites were significantly affected by the medium carbon source.

### Optimization of the Growth Conditions in Bench-Top Fermenter

In contrast with previous findings obtained with *Enterobacter* and *Agrobacterium*, in which the *ipdC* gene is under the control of TyrR and the accumulation of IAA occurred only after entrance in the stationary phase (Ryu and Patten, 2008), when *P. agglomerans* strain C1 was grown on LB medium, as well as on M9 glucose, no significant production of IAA was detected when Trp was provided to resting cells or to stationary-phase cultures. To better understand the link between the cell physiological state and the biosynthesis of IAA in *P. agglomerans*, strain C1 was grown under controlled bioreactor conditions ensuring optimum oxygen uptake and temperature control and high growth rates.

**FIGURE 8:**
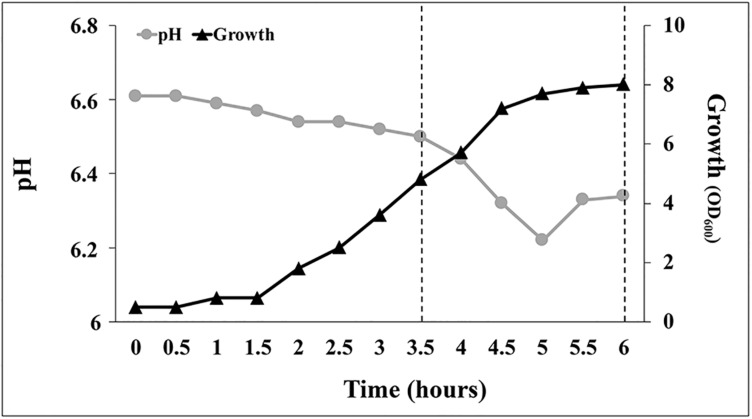
Growth pattern and pH evolution during batch scale tests in YES medium under aerobic conditions at 30°C. Data points are mean values of duplicate fermentations.

Analyzing the growth profile of C1 cultures grown at 30°C on YES medium under aerobic conditions (DO level > 20% of saturation), a lag phase of 30 min was observed followed by an acceleration phase (up to 1.5 h) during which the growth rate gradually increased (Figure 8). In the exponential phase, which reached its maximum at approximately 4 h, the specific growth rate was 1.63 h^–1^. Between the 4th and the 5.5th hour, the specific growth rate decreased to approximately 67% (from 1.63 to 0.53 h^–1^) and at approximately 5.5 h the culture entered in the stationary phase (Figure 8). Interestingly, during the first 3.5 h of growth, medium pH decreased slowly from 6.6 to 6.5, at a rate of approximately 0.028 unit per hour (Figure 8). In YES medium, when the culture reached the end of the exponentially phase (4 h), the pH started to decrease rapidly (at a rate of 0.87 unit per hour) and reached a minimum value of 6.22 at 5 h. Later, at the beginning of the stationary phase, the medium pH increased from 6.22 up to 6.34 and remained constant up to the end of the fermentation (Figure 8). Variations in the medium pH between the 3.5th and the 5.5th hour occurred concurrently with changes in the oxygen consumption rate that were controlled by DO-dependent modifications of the agitation speed and the airflow using a closed-loop system. As shown in Supplementary Figures S1B,C, the oxygen demand suddenly increased between 4 and 4.5 h (agitation speed increased > 10% in 30 min, from 400 to 450 rpm) and then remained stable up the beginning of the stationary phase (5.5 h).

The growth of *P. agglomerans* strain C1 in bioreactor allowed increasing the biomass yield compared to shake-flask cultures of about sixfold, up to 292.5 ± 4.8 g [wet weight] L^–1^.

### Influence of the Physiological State of the Inoculum on IAA Production

**FIGURE 9:**
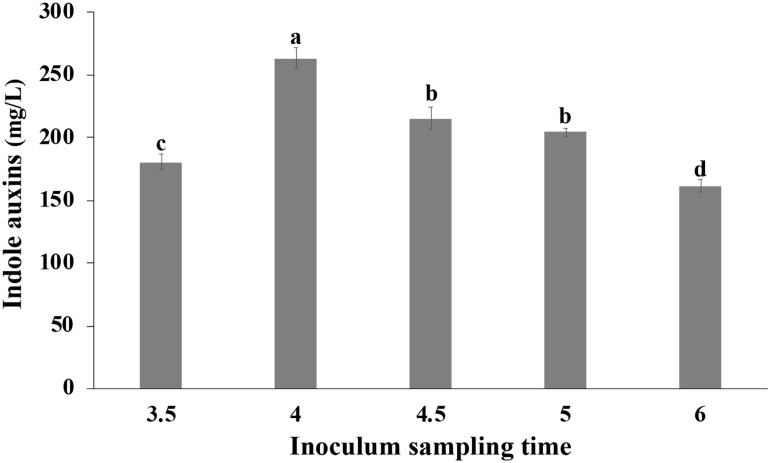
Effect of the physiological state of the cells used for inoculation on IAA production. *Pantoea agglomerans* strain C1 was cultivated on YES-tryptophan medium for 18 h, and cells used for the inoculum (OD = 0.5) were collected by the fermenter at the time points indicated on the *x*-axis. Differences in letters indicate that the values are significantly different (*P* > 0.05).

Cells collected at different time points during the growth in bioreactor were transferred in medium containing Trp, so as to evaluate the effect of the physiological state of the cells used for inoculation on the IAA production. The growth was carried out in shake flasks at 30°C, and the IAA production was monitored during the first 2 h and at the end of the growth. In all tested conditions, no significant difference was observed in the initial rate of IAA production that was approximately 42 ± 2 g L^–1^ h^–1^. Surprisingly, at the end of the growth, a significant influence of the inoculum was observed on the IAA titer that varied between 161.58 ± 4.91 (6 h old inoculum) and 263.33 ± 8.25 mg/L (4 h old inoculum; Figure 9).

### Use of IAA-Enriched Excretome From *P. agglomerans* C1 in Plant Nursery

The efficacy of the exhausted culture medium, containing IAA and other secreted metabolites from *P. agglomerans* strain C1, named IAA-E_C__1_ (for IAA-enriched Excretome from strain C1) was tested on a total of 4,540 plants of *Prunus* rootstock GF/677 and 1,080 plants of hazelnut cv. Fertile de Coutard. The experiments were carried out in a large-scale nursery farm (Vivai Piante Battistini, Cesena, Italy) according to the protocols used for large agamic propagation of fruit crop trees.

### Plant Survival Experiments

The inductive activity of adventitious rooting of exometabolites from strain C1 was tested *in vitro* on two sets of 760 plantlets of rootstock GF677 treated with either a 3 μM IBA-K solution or an appropriate volume of IAA-E_C__1_ to achieve a final concentration of auxins of 1 μM. After 10 days of rooting stage, when plantlets were transferred to 360-cell plug tray, the percentage of survival was 85% for IAA-E_C__1_–treated plants and 97% for IBA-treated plants; after 1 month of *ex vitro* acclimation, this value resulted 95% for IAA-E_C__1_–treated plants and only 80% for IBA-treated plants. Taken together, these values indicate an overall increase of about 3% in plant survival when bacterial metabolites were used in alternative to IBA-K.

### *Ex vitro* Experiments

**FIGURE 10:**
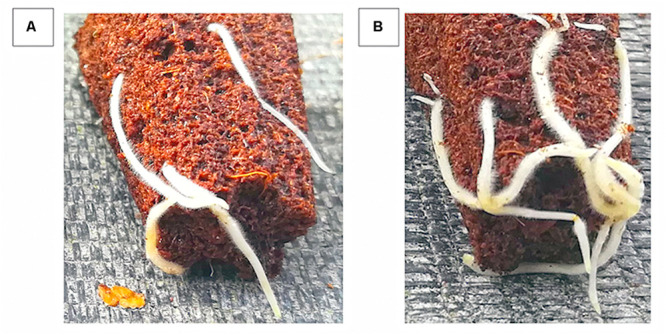
Effect of different auxin treatments on morphology of rootstock GF677 roots. **(A)** IBA-K (3 μM); **(B)** diluted IAA-E_C1_ solution (IAA = 1 μM).

After 20 days of *ex vitro* acclimatization, regardless of the inductive treatments, the percentage of rooting was 100%. However, the number of roots per rooted explant was significantly higher for IAA-E_C__1_–treated plants (5.2 ± 0.5 roots per explant), compared to an average of 3.6 ± 0.5 of roots for IBA-K–treated plants. The quality of the roots also resulted improved after the treatment with IAA-EC (Figure 10). No significant difference was, instead, detected for the elongation of roots, which was approximately 14 mm for all plants, as well as for the plant survival percentage that, after 3 weeks from the beginning of the experiments, was 85% ± 1%. Finally, the average total leaf area per plant was higher in IAA-E_C__1_–treated (115 ± 2 mm^2^) with respect to IBA-K–treated plants (102 ± 8 mm^2^).

**FIGURE 11:**
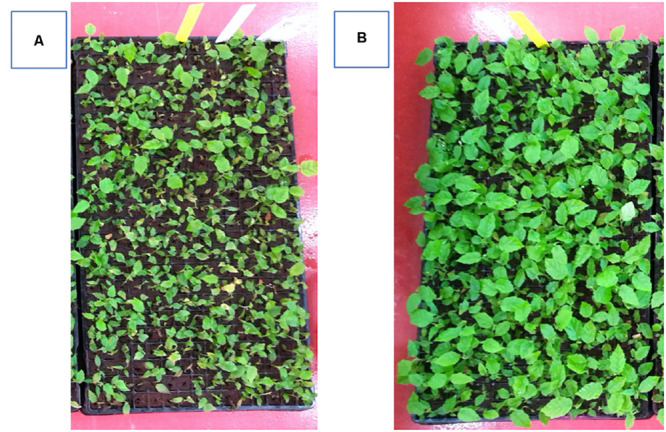
Effect of different auxin treatments on plant growth of *Corylus avellana* L. cv. “Fertile de Coutard.” **(A)** IBA-K (3 μM); **(B)** diluted IAA-E_C1_ solution (IAA = 1 μM).

**FIGURE 12:**
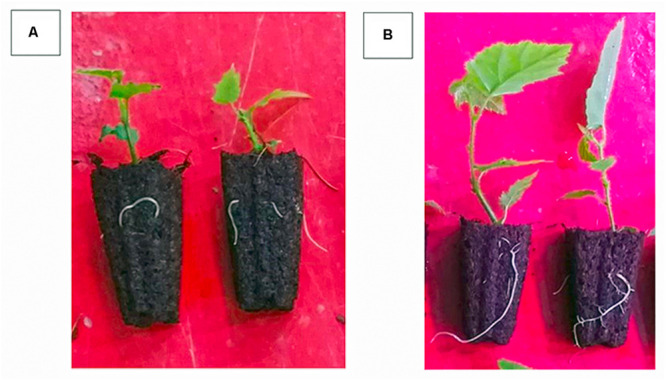
Effect of different auxin treatments on morphology of *Corylus avellana* L. cv. “Fertile de Coutard” roots. **(A)** IBA-K (3 μM); **(B)** diluted IAA-E_C1_ solution (IAA = 1 μM).

The inductive role of adventitious rooting by IAA-E_C__1_ was also investigated on hazelnut microcuttings of cv. Fertile de Coutard using the same experimental protocol applied for *Prunus* rootstock. This experiment was carried out on two distinct pools of 1,080 and 1,440 binodal cuttings that were treated with IBA-K (3 μM solution) and IAA-E_C__1_ (a 1:1000 diluted solution with an indole auxins final concentration of 1 μM), respectively. The percentage of rooted cuttings and number of roots per rooted explants resulted to be not significantly affected by the treatments, with the rooted ratio being between 64 and 75% and the value of root number being between 1.1 ± 0.1 and 1.3 ± 0.2 (Table 2). The root length was significantly affected by C1 metabolites and increased about 1.3-fold after treatment with IAA-E_C__1_ compared to IBA-K (Table 2). A similar positive effect was also observed analyzing the stem elongation and the leaf area which both increase of about 1.4-fold in IAA-E_C__1_–treated plants (Figure 11). It is worth mentioning that the treatment with C1 metabolites determined a development of adventitious roots that are not present in IBA-K–treated plants (Figure 12).

**TABLE 2:** Effect of different auxin treatments on plant length, leaf area, number of roots per cutting, and root length of hazelnut cv. “Fertile de Coutard.”

**Treatment**	**Plant length (cm)**	**Leaf area (cm^2^)**	**No. roots per cutting**	**Root length (cm)**
IBA-K (3 μM)	17.8 ± 1.5^b^	164 ± 5^b^	1.3 ± 0.2^a^	9.4 ± 0.5^b^
IAA-E_C__1_ (1 μM*)	25.7 ± 1.7^a^	234 ± 5^a^	1.1 ± 0.1^a^	12.2 ± 0.5^a^

Means followed by the same letter were not significantly different (P < 0.05). *IAA conc.

## Discussion

Indole-3-acetic acid production is widespread among plant growth–promoting bacteria and varies from species to species and also among strains belonging to the same species (Spaepen and Vanderleyden, 2011; Duca et al., 2014). Auxins and other plant growth–promoting metabolites obtained from non-pathogenic *P. agglomerans* strains can be successfully applied in agriculture systems as plant biostimulants. It has been demonstrated that *P. agglomerans* strain C1 produces IAA and siderophores, and the metabolites secreted by this strain can be utilized as biostimulants to improve the root surface area in tomato cuttings (Luziatelli et al., 2020). This strain is also able to solubilize phosphates and can improve the use of rock phosphates by corn (*Zea mays* L.) and tomato (*S. lycopersicum* L.) (Saia et al., 2020).

The application of comparative genetics, metabolomics, and fermentation technology in this study allowed analyzing in more detail the possibility of using this strain for production of a novel cell-free biostimulants. Whole-genome analysis demonstrated that *P. agglomerans* C1 has several genes connected with the production of IAA from Trp and all the genes of the IPyA pathway (Table 1 and Figure 1). In GenBank, there are several sequences encoding indolepyruvate decarboxylase, the key enzyme of the IPyA pathway, and homologous enzymes, but only in few cases that the corresponding proteins have been isolated and characterized for their biochemical properties. The 1,653 bp peg1955 gene from *P. agglomerans* C1 encodes a 551-amino acid protein, which shares high identity with well-characterized IPDC from other *Pantoea* strains: 92% identity with the IPDC from *P. agglomerans* 299R (Brandl and Lindow, 1996) and 73% identity with IPDC1 from *Pantoea* species YR343 (Garcia et al., 2019; Table 1 and Figure 2). Interestingly, the results obtained in this study demonstrated that, with few exceptions, in most of the sequenced genomes of isolates belonging to *P. agglomerans* species (45 of 50), the genes encoding enzymes of the IPyA pathway share an identity higher than 95% over the full-length of the sequence (Figure 4). This unusually high sequence conservation among bacteria occurring in diverse natural environments has never been reported before for IAA genes belonging to other species. This is a strong evidence of the evolutionary importance of this functional trait for the interaction between *P. agglomerans* and host plants.

Molecular data presented in this work also indicate that strain C1 produces IAA through the IPyA route, which is usually associated to beneficial bacteria, whereas the IAM pathway, linked to plant pathogens using auxin synthesis as a virulence factor (Patten et al., 2013), is not present. These observations encourage the exploitation of strain C1 and its metabolites as a plant growth promoter.

Interestingly, comparative genomics also allowed demonstrating that the non-coding region upstream of the *ipdC* gene is highly conserved among member of *P. agglomerans* species (Figure 4) and that this gene is not regulated by TyrR (Figures 3, 5). TyrR is a transcriptional regulator that controls the expression of a number of genes involved in the biosynthesis, catabolism, and transport of aromatic amino acids in *E. coli* (Pittard et al., 2005) and regulates expression of *ipdC* gene in *E. ludwigii* UW5 (Coulson and Patten, 2015). The *ipdC* promoter from strain UW5 contains two TyrR binding motifs, a strong (highly conserved) and a weak Tyr-box, which are not present in *Pantoea* strains (Figure 5). This result agrees with the observation that in *P. agglomerans* C1 the IAA production occurs in exponential phase of growth and not in stationary phase as in species in which *ipdC* gene is under the control of TyrR. The regulatory mechanism that control expression of the genes involved in IPyA pathway in *P. agglomerans* has not been elucidated yet, although there is evidence that multiple regulatory mechanisms may exist. For example, Brandl and Lindow (1997) demonstrated that in *P. agglomerans* strain 299R *ipdC* gene was expressed at low levels when cells were cultured in *in vitro* liquid cultures (independently from the presence of Trp or the growth phase of the culture) and was fully induced only when cells were grown on plants under water stress. In contrast, IAA production in *P. agglomerans* C1 is Trp-dependent and is significantly affected from the carbon source (Figure 6) and the physiological state of the cells (Figure 9). Moreover, cultivation in bench-top fermenter indicated that with strain C1 there is a correlation between the amount of IAA produced after induction with Trp and the preparation of the inoculum (Figure 9). This observation agrees with the findings of a previous work with recombinant *E. coli* cells, where the production of aromatic compounds in enteric bacteria is enhanced with cells in exponential phase of growth in which the availability of ATP is higher (Luziatelli et al., 2019c).

Untargeted metabolomics allowed demonstrating that *P. agglomerans* C1, on medium containing sucrose as a carbon source, produces, together with IAA, other IAA-related compounds, such as I3C and IAA-leucine, which can either modulate the effect of an excess of IAA (Katz et al., 2015) or increase the availability of this compound (Sanchez Carranza et al., 2016). In the exometabolome of *P. agglomerans* C1, other classes of compounds are also present, such as peptides and cyclopeptides that crosstalk with auxins or affect auxin transport or turnover.

Finally, the results obtained from studies run on a large scale on both one of the most diffuse *Prunus* rootstock and plant hazelnut confirmed that metabolites secreted by indole auxin–producing cells of *P. agglomerans* C1 have a high stimulating effect on adventitious rooting induction and adaption and plant growth *in vitro*. Interestingly, the excretome of strain C1 can also play relevant roles in root morphology (Figures 10, 12) and plant growth (Figure 11) in *ex vitro* conditions. It is worth noting that these effects, as shown by the control experiments with IBA-K, are only in part dependent on auxins and with IAA-E_C__1_ can be achieved at a molar concentration of IAA_equ_ threefold lower than the synthetic auxin (1 vs. 3 μM). Both *in vitro* and *extra vitro* procedures have shown that IAA-E_C__1_ improved the performance and quality of micropropagated plant production. It should be emphasized that evaluation of the applicability of *Pantoea* metabolites in plant nursery industry was carried out following standard operation procedures used for large-scale production. Best practices for plant production rely on a careful control of microbial contamination of the production lines and equipment, the growing media, and the plant containers. In this specific context, the use of microbial inocula that can be beneficial for some plant cultures and not appropriate for others is always questionable and potentially dangerous. Under this respect, the use of liquid products, which are cell-free and contain only microbial metabolites, reduces contamination risks, is more compatible with the automation systems, and facilitates the adoption of this technology in plant nurseries.

## Conclusion

In conclusion, the use of metabolites secreted by selected strains of *P. agglomerans* species can provide a valuable contribute for production of innovative biostimulants that can comply with current EU legislation. Further studies on gene expression will help to decipher the regulatory network that control IAA biosynthesis in *P. agglomerans* and provide more insight into the mechanisms by which *Pantoea* metabolites elicit plant growth promotion.

## Data Availability Statement

All datasets presented in this study are included in the article/Supplementary Material.

## Author Contributions

FL, AF, PB, RM, and MR contributed to the conception and design of the study and wrote the first draft of the manuscript. FL, AF, PB, LG, RM, MM, MT, and MR contributed to define the methodology. FL, AF, PB, and MR contributed to software analysis and data validation. FL, AF, PB, LG, RM, MM, MT, FM, and MR contributed to investigation. FL, AF, RM, FM, and MR wrote and edited the final version of the manuscript. All authors have read and agreed to the published version of the manuscript.

## Conflict of Interest

PB was employed by Next-Generation Agronomics Laboratory. MM was employed by the company Vivai Piante Battistini soc. agr. s.s.

The remaining authors declare that the research was conducted in the absence of any commercial or financial relationships that could be construed as a potential conflict of interest.

## Footnotes

1 http://www.softberry.com/berry.phtml?topic=bprom&group=programs&subgroup=gfindb

2 https://patricbrc.org/

3 www.canopeoapp.com
