# Supplementary material for: A Genetic and Metabolomic Perspective on the Production of Indole-3-Acetic Acid by Pantoea agglomerans and Use of Their Metabolites as Biostimulants in Plant Nurseries
Source: Front Microbiol. 2020 Jul 14;11:1475. doi: 10.3389/fmicb.2020.01475 (PMC7381177; doi:10.3389/fmicb.2020.01475)

A Genetic and Metabolomic Perspective on the Production of Indole-3-Acetic Acid by *Pantoea agglomerans* and Use of Their Metabolites as Biostimulants in Plant Nurseries

Francesca Luziatelli[1]†, Anna Grazia Ficca[1]†, Paolo Bonini[2], Rosario Muleo[3], Lorenzo Gatti[3], Massimiliano Meneghini[4], Michele Tronati[1], Francesca Melini[1,5], and Maurizio Ruzzi[1*]

Supplementary Material

**TABLE S1 | ** Results of the ChemRICH enrichment analysis for the altered metabolites secreted by *P. agglomerans* C1 shifting from glucose (YEG) to sucrose (YES). Data are referred to increased/decreased levels when sucrose was provided as a carbon source.

**Cluster name**	**Cluster size**	**p-values**	**FDR**	**Altered metabolites**	**Increased**	**Decreased**
NewCluster_1	23	3,5E-19	3,4E-18	23	21	2
Peptides, Cyclic	22	1,9E-18	1,6E-17	22	21	1
Indoles	18	2,2E-20	2,6E-19	18	10	8
NewCluster_3	14	1E-12	3,4E-12	14	12	2
Dipeptides	11	2,2E-20	2,6E-19	11	9	2
NewCluster_21	10	2,2E-20	2,6E-19	10	10	0
NewCluster_49	9	2,2E-20	2,6E-19	9	9	0
Triterpenes	9	2,2E-20	2,6E-19	9	9	0
Isoquinolines	9	0,000000021	0,000000032	9	8	1
Saponins	8	1,1E-14	4,7E-14	8	8	0
NewCluster_35	7	2,9E-10	5,9E-10	7	7	0
Diterpenes	6	1,5E-14	5,7E-14	6	5	1
Alkaloids	6	7,9E-12	2,3E-11	6	5	1
Oligopeptides	6	3,9E-09	6,9E-09	6	6	0
Indole Alkaloids	6	0,000000012	0,000000019	6	5	1
Sesquiterpenes	6	0,000000015	0,000000023	6	4	2
Cevanes	6	0,0000062	0,0000067	6	6	0
Cholestenones	5	1,1E-16	5,9E-16	5	5	0
Depsipeptides	5	1,1E-16	5,9E-16	5	5	0
Indans	5	1,1E-16	5,9E-16	5	5	0
SRS-A	5	1,1E-16	5,9E-16	5	5	0
Aminoglycosides	5	1,2E-15	5,9E-15	5	5	0
NewCluster_47	5	2,3E-15	1E-14	5	5	0
Pyrrolizidine Alkaloids	5	6,4E-14	2,3E-13	5	3	2
Azoles	5	2,4E-12	7,4E-12	5	3	2
Disaccharides	5	9,8E-12	2,7E-11	5	3	2
Flavonoids	5	3,6E-11	9E-11	5	2	3
Cinnamates	5	2,1E-10	4,3E-10	5	4	1
Coumarins	5	1,7E-09	3,1E-09	5	3	2
Morphinans	5	0,000000039	0,000000058	5	5	0
Glycerophospholipids	5	0,0000002	0,00000026	5	5	0
Quaternary Ammonium Compounds	4	7,2E-14	2,5E-13	4	4	0
Phenylurea Compounds	4	1,1E-11	2,8E-11	4	4	0
Purinones	4	8,1E-11	2E-10	4	2	2
Kanamycin	4	1,2E-10	2,6E-10	4	4	0
NewCluster_4	4	5,7E-09	9,7E-09	4	1	3
Amino Acids	4	0,0000055	0,000006	4	3	1
Peptides	3	8,4E-11	2E-10	3	2	1
Withanolides	3	1,6E-10	3,3E-10	3	3	0
Secologanin Tryptamine Alkaloids	3	3,5E-10	6,8E-10	3	3	0
Pregnanes	3	7E-10	1,3E-09	3	3	0
Oxazoles	3	6,3E-09	0,000000011	3	3	0
Berberine Alkaloids	3	0,000000047	0,000000068	3	2	1
Lactones	3	0,000000085	0,00000012	3	2	1
Glucosides	3	0,0000001	0,00000014	3	3	0
Furans	3	0,00000011	0,00000015	3	3	0
Quinolines	3	0,00000015	0,00000019	3	2	1
Pyrimidinones	3	0,00000036	0,00000046	3	2	1
Carbolines	3	0,00000059	0,00000072	3	2	1
Stilbenes	3	0,00000059	0,00000072	3	1	2
Succinates	3	0,00000069	0,00000081	3	1	2
Pyridines	3	0,00000078	0,0000009	3	2	1
Pyrans	3	0,00000088	0,000001	3	2	1
Glycosides	3	0,00000095	0,0000011	3	3	0
Heterocyclic Compounds, 3-Ring	3	0,000019	0,00002	3	3	0
beta-Lactams	3	0,000021	0,000022	3	1	2
Cyclohexanes	3	0,000044	0,000045	3	3	0
Naphthalenes	3	0,000097	0,000097	3	2	1

TABLE S2 | List of indoles and IAA-aa metabolites found to be significantly different shifting from glucose (YEG) to sucrose (YES).

**Compound name**	**PubChem ID**	**P value**	**foldchange**	**Cluster number**	**xlogP**	**ChemRICH Cluster**	**FDR**
Indole-3-carbinol	3712	0,00038	4.3	53	0.996	Indoles	0.13
Indole-3-acetic acid	802	0,0087	2.2	53	0.805	Indoles	0.8
Indole-3-carboxaldehyde	10256	0,0002	0.36	53	1.466	Indoles	0.098
Indoleacetaldehyde	800	0,00042	0.34	53	1.219	Indoles	0.13
Indole-3-acetyl-L-valine	5165230	0,0019	39	4	1.158	Oligopeptides	0.37
Indole-3-acetyl-L-leucine	688142	0,0066	2.4	4	1.938	Oligopeptides	0.77

FIGURE S1 | Temporal changes in DO concentration, stirred speed, and airflow during cultivation of *P. agglomerans* C1 on YES medium in a 7.5-L stirred tank reactor at 30°C in unbuffered conditions.

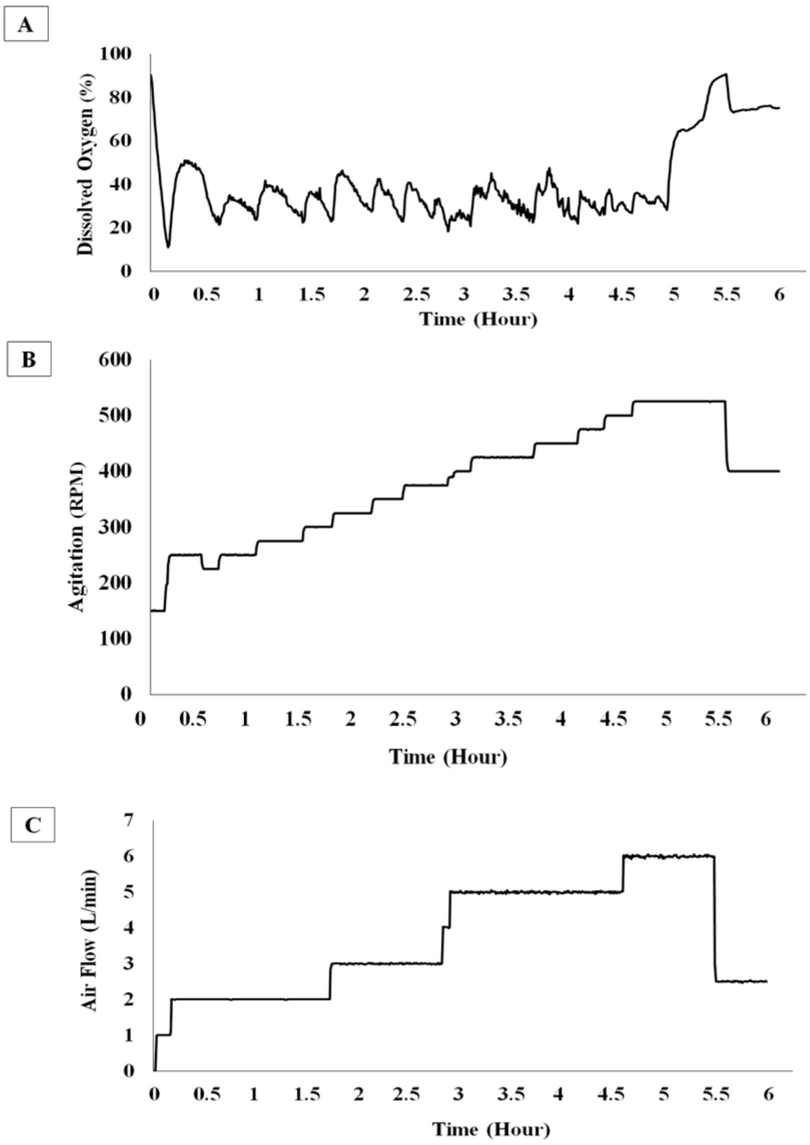

Supplement: Supplementary file 1 [file Data_Sheet_1.PDF]
